# Supplementary material for: Witnessing the onset of reionization through Lyman-α emission at redshift 13
Source: Nature. 2025 Mar 26;639(8056):897–901. doi: 10.1038/s41586-025-08779-5 (PMC11946913; doi:10.1038/s41586-025-08779-5)
Supplement: Supplementary file 1 — Supplementary Information, including Supplementary Figures 1–5, Supplementary Table 1 and further references. [file 41586_2025_8779_MOESM1_ESM.pdf]

nature portfolio

https://doi.org/10.1038/s41586-025-08779-5

Supplementary information

Witnessing the onset of reionization through Lyman-α emission at redshift 13

In the format provided by the authors and unedited

Supplementary information

NIRCam imaging

The NIRCam imaging data set in the JOF comprises 14-band imaging of uniform depth, each having 5σ limits below $\sim$ 3 nJy for a point source[14,16]. A false-colour image centred on JADES-GS-z13-1-LA created from stacked NIRCam image mosaics is shown in Supplementary Material Fig. 1. The blue channel is the mean of the F090W and F115W filters, green similarly combines F150W and F162M, while red is a stack of F182M, F200W, and F210M (panel d of Fig. 1 was created similarly with all available filters). Photometric redshifts of nearby galaxies have been derived with EAZY[113] as outlined in Hainline et al.[15].

NIRSpec data reduction

We considered various reduction variations, including one that reduces self-subtraction in cases where the emission is extended over multiple micro-shutters by only considering the two outer nod positions instead of the default three. We have verified that the differences between this reduction and the default one considering all nod positions are minimal, including for the emission line observed at $\lambda_{\rm obs} \approx 1.7\,\mu$m. The same is true for reduction variants where one-dimensional spectra are extracted over the central three or five spatial pixels (corresponding to $0.3''$ or $0.5''$ respectively), as expected given the compactness of JADES-GS-z13-1-LA (Methods). We therefore base our analysis on 3-pixel extractions considering all three nodding positions to maximise the SNR. Additional path losses are accounted for through a comparison with NIRCam photometry, as discussed in the Methods.

The pipeline independently produces two- and one-dimensional spectra from all reduced sub-exposures, of which there are a substantial number for JADES-GS-z13-1-LA (48 for the PRISM and 12 for each of the R1000 gratings). Since cosmic ray impacts and noisy pixels are not always fully captured in the ramp fitting[61] and occasionally introduce a sharp excess of flux in these individual sub-exposures, the standard reduction pipeline adopts iterative sigma clipping[56] for each wavelength bin before co-adding the reduced one-dimensional spectra. This combination process weights individual sub-spectra inversely by the pipeline error estimate squared. The combination of two-dimensional spectra is performed separately[56], and therefore the final combined two-dimensional spectrum (as shown in Fig. 1) is not used to directly extract one-dimensional spectra. Recently, however, several refinements to this standard procedure were presented in a detailed investigation[9] (see also ref.[114]) of ultra-deep NIRSpec/PRISM measurements of two $z > 10$ galaxies benefiting from a large number of sub-exposures (186 and 138 respectively), which we follow here to obtain our fiducial ('sigma-clipped') spectrum.

Specifically, Hainline et al.[9] introduced two additional masking steps aimed at removing obviously spurious flux values at the beginning of the combination process. In each step, the median flux value and median pipeline error estimate for each wavelength bin are calculated across available sub-exposures. Wavelength bins of sub-spectra where flux values deviate by more than five times the median error from the median flux, or where the pipeline error estimate exceeds five times its median, were eliminated. Subsequently, five iterations of sigma clipping removed flux values deviating from the average of the surviving entries in the bin by more than three times the sample standard deviation. Finally, the individual sub-spectra were co-added simply by averaging the surviving entries in each wavelength bin. As in Hainline et al.[9] and Curti et al.[114], we constructed 2000 bootstrapped realisations of the final combined spectrum from which we derived a covariance matrix. Crucially, this covariance matrix directly captures all possible sources of statistical fluctuations present in the data (including those not explicitly taken into account by the default pipeline) as well as the significant degree of correlation in the noise of adjacent wavelength bins.

We note that while the intra-shutter location of JADES-GS-z13-1-LA is nearly identical across the two visits (Fig. 1), the sub-exposures follow a three-point nodding pattern, and the employed micro-shutters differ by one column between the two visits (quadrant 3, row 237, column 87 versus quadrant 3, row 237, column 86). As a result, the two-dimensional spectra, including the emission line at $\lambda_{\rm obs} \approx 1.7\,\mu$m, are shifted vertically by $\sim$ 15 pixels on the detector over the range of the 48 sub-exposures, thus minimising the potential impact of bad pixels. We have performed careful visual inspection of the raw NIRSpec exposure frames to verify the spectrum does not suffer from overlapping targets or contamination from other (failed) open micro-shutters. Finally, there are no signs of hot pixels or significant particle hits affecting the line at $\lambda_{\rm obs} \approx 1.7\,\mu$m in the extracted spectrum: in the four wavelength bins where the line appears in the combined PRISM spectrum, the refined sigma-clipping algorithm described above has classified 46 out of 48 values as valid for one bin, while all 48 values in the remaining three bins were deemed valid.

NIRSpec/PRISM spectra

In Supplementary Material Fig. 2, we compare different one-dimensional PRISM spectra of JADES-GS-z13-1-LA, including our fiducial spectrum, obtained from the refined sigma-clipping procedure applied on 3-pixel extractions of all available sub-exposures (NIRSpec data reduction), and the default STScI pipeline spectrum. From this comparison, we conclude these generally agree very well, although a number of noise spikes can be seen in the STScI spectrum. We also show spectra obtained separately from the two visits, which both show good agreement with the combined spectrum, including for the emission line observed at $\lambda_{\rm obs} \approx 1.7\,\mu$m.

Furthermore, we provide a comparison with the NIRSpec/PRISM spectrum of GS-9422 at $z = 5.94$ (JADES-GS+53.12175-27.79763 in Bunker et al.[56]; see also refs.[44, 74, 115]), whose spectrum simultaneously displays strong Lyα emission, a Balmer jump, and a steep turnover seen in the UV (rest-frame wavelengths below $\lambda_{\rm emit} \lesssim 1500$ Å). To guide the eye, Supplementary Material Fig. 2 shows the 2γ continuum for gas at electron density of $n_e = 10^3\,{\rm cm^{-3}}$ and electron temperature of $T_e = 20\,000$ K (though we note that unlike the normalisation, the shape of the 2γ continuum does not depend on these conditions[75]), which, like the spectrum of GS-9422, is shifted in wavelength to $z = 13$ and rescaled to match the observed flux density of JADES-GS-z13-1-LA at $\lambda_{\rm emit} = 1500$ Å.

A detailed view of the PRISM wavelength bins near the emission line observed at $\lambda_{\rm obs} \approx 1.7\,\mu$m is shown in Supplementary Material Fig. 3. We performed jackknife resampling of all sub-spectra to independently estimate the uncertainty on the mean flux measured in each wavelength bin across all sub-exposures (in the absence of any masking). These are in excellent agreement the fiducial, sigma-clipped spectrum and uncertainty from the bootstrapped covariance matrix (NIRSpec data reduction), suggesting the measurements are regularly distributed without extreme outliers. We also computed the (unmasked) integrated flux across four bins with SNR > 1 for each sub-spectrum separately. The resulting SNR = 6.4 on the integrated flux estimated using jackknife resampling again agrees well with the SNR estimated via the sigma-clipped spectrum and corresponding bootstrapped covariance matrix, as will be discussed in Emission-

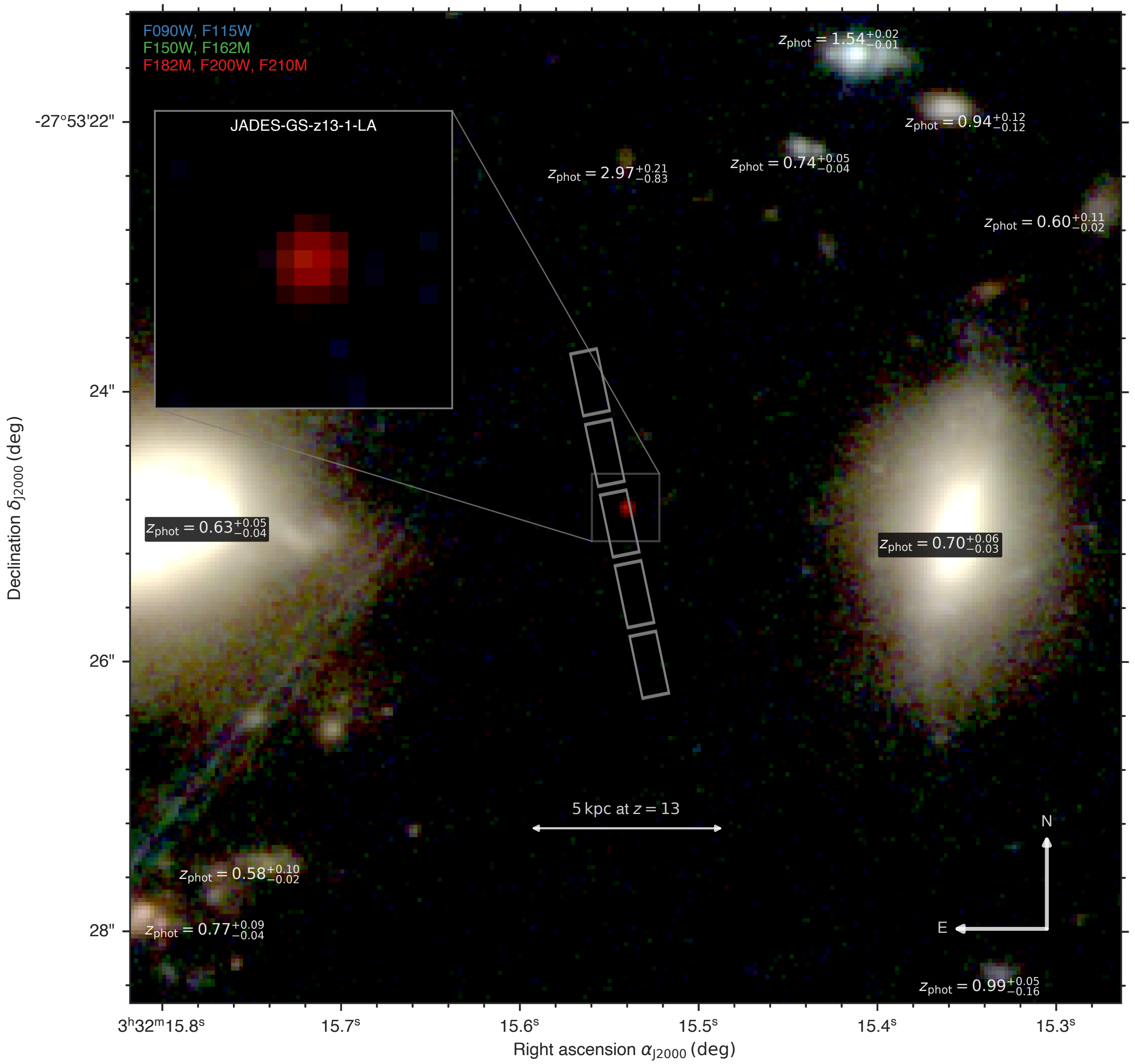

Supplementary Material Fig. 1 | NIRCam false-colour image of JADES-GS-z13-1-LA and its surroundings. The image shows stacks of the F090W and F115W filters as the blue channel, the F150W and F162M filters as green, and F182M, F200W, and F210M as red. To the bottom left, a diffraction spike can be seen stretching diagonally across the image, coming from a star located just outside the field of view. Annotations show the photometric redshifts of nearby sources, including two extended low-redshift galaxies. The placement of the NIRSpec micro-shutters is shown in grey. At the bottom centre, a scale of 5 kpc at $z = 13.0$ is indicated.

line properties. The STScI reduction, on the other hand, nominally returns a higher significance for the line detection (SNR = 8.5), likely reflecting a slight underestimation of the (correlated) noise (NIRSpec data reduction).

We consider here whether the emission line at 1.71 µm may be due to contamination of the micro-shutter by a foreground source that is aligned with JADES-GS-z13-1-LA by chance and remains undetected in the continuum. Firstly, such a low-redshift interloper must have an exceptionally high EW to respect the stringent non-detections in the bluest NIRCam filters (e.g. the F150W 3σ constraint implies an observed EW $\gtrsim 10\,000$ Å if the continuum were flat in F_ν). Therefore, the line could realistically only be Hα at $z \approx 1.60$, where Paα from the same foreground source would be observed at 4.88 µm, or [O III] $\lambda\,5008$ Å at $z \approx 2.41$ where the Hα and [N II] complex would fall at 2.24 µm. The absence of any accompanying line detections in the first case implies Hα/Paα > 13.8 (2σ), in tension with the case-B ratio[91] of Hα/Paα ≈ 10 which can only be decreased by dust attenuation. Alternatively, for the interloper to be at $z \approx 2.41$ requires a line ratio ([O III]/(Hα + [N II]) > 7.7 at 2σ) completely incompatible with observed properties of $z \sim 2$ galaxies[116]. Leaving

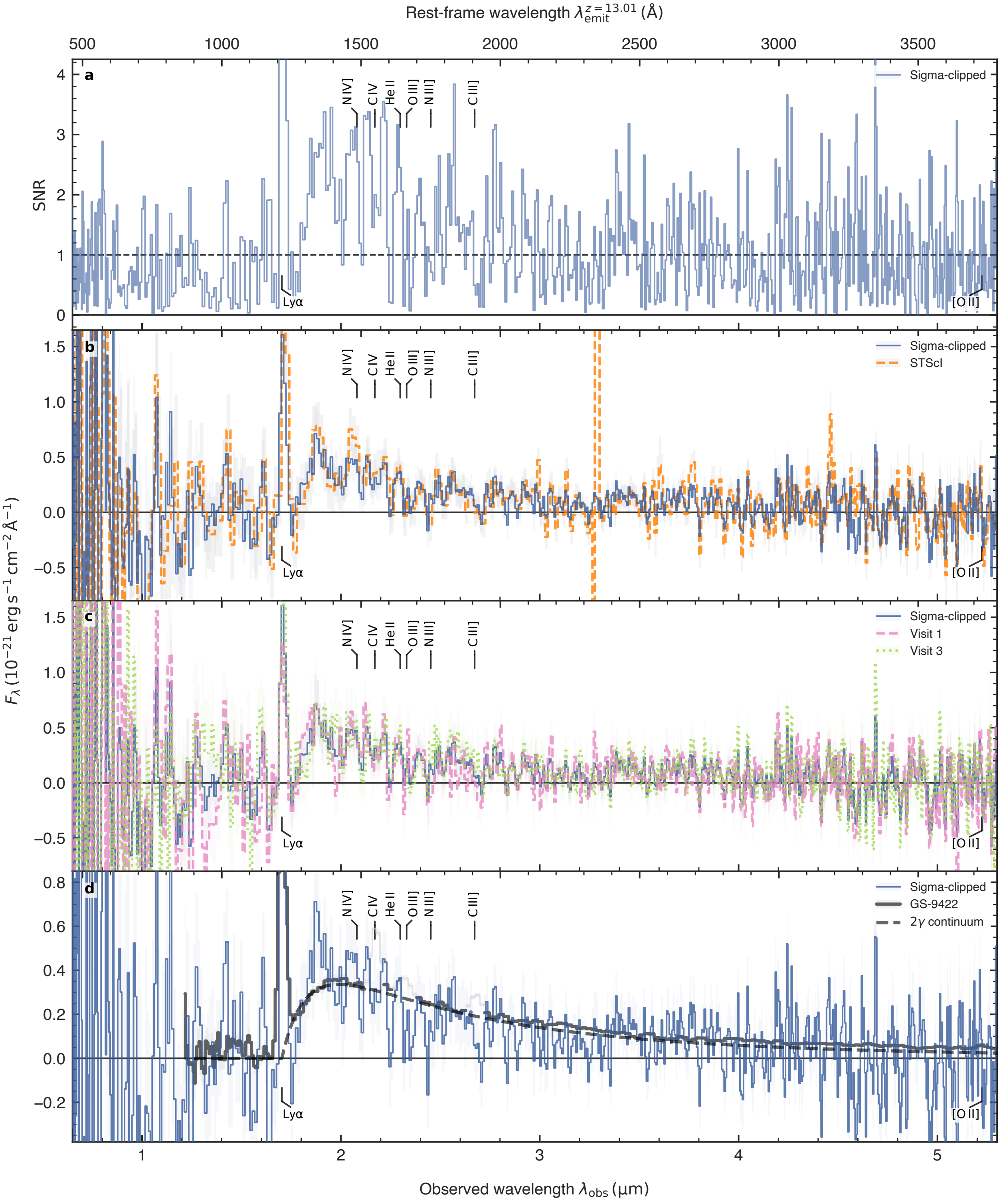

Supplementary Material Fig. 2 | Comparison of NIRSpec/PRISM spectra of JADES-GS-z13-1-LA. a, SNR on individual wavelength bins for the sigma-clipped spectrum combining all sub-exposures (also displayed in other panels). The uncertainty on individual spectral bins considered here only represents diagonal elements of the covariance matrix. **b**, Comparison with the default STScI pipeline reduction (see NIRSpec observations and data reduction for details). **c**, Comparison with spectra of the two visits separately. **d**, Comparison of the spectrum of JADES-GS-z13-1-LA with that of GS-9422 (strong UV lines greyed out to ease comparison) and the two-photon (2γ) continuum (both rescaled; see text for details). Shading in panels b through d shows 1σ uncertainty. All panels show the expected locations of key UV emission lines: Lyα (detected), N IV], C IV, He II, O III], N III], C III], and [O II] (all undetected).

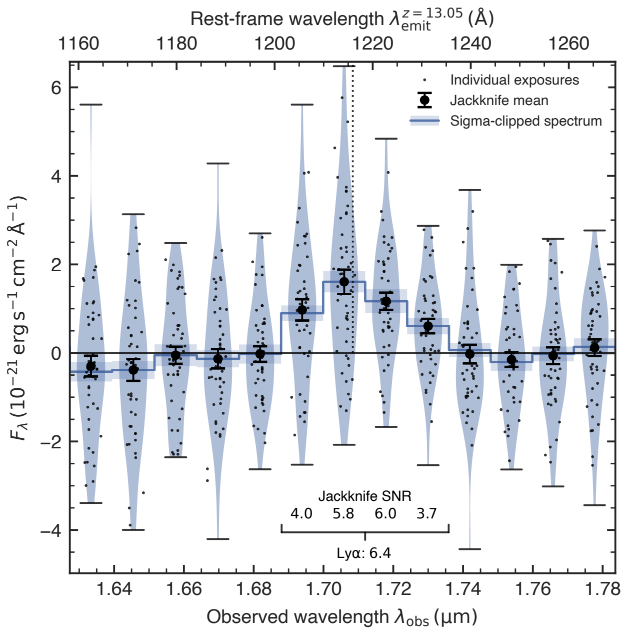

Supplementary Material Fig. 3 | Individual sub-spectra of JADES-GS-z13-1-LA around 1.7 μm**.** Our fiducial, sigma-clipped spectrum is shown by the step-wise blue line, with shading representing the 1σ uncertainty for each individual spectral bin as retrieved from the diagonal element of the covariance matrix. The flux values extracted from individual sub-exposures are shown as small black circles within each wavelength bin (slightly offset in the wavelength direction for visualisation). Their distribution is illustrated with a kernel-density estimate shown by a blue swath, enclosed by short horizontal black bars indicating the flux extrema. Larger black circles represent their mean, with errorbars showing the 68% confidence interval estimated via jackknife resampling. The jackknife SNR is annotated for four individual bins with SNR > 1, as well as for the combined flux across these four bins.

aside EW arguments, for the line to be the [O ii] λ 3727, 3730 Å doublet ([O ii]) at $z \approx 3.58$, similarly unprecedented line ratios would be required ([O ii]/Hβ > 8.4 and [O iii]/[O ii] < 0.11 at 2σ).

We conclude the only consistent explanation is that the line is Lyα at $z \approx 13$. However, we note its observed properties also cannot be due to the extended Lyα photon-diffusion emission predicted to surround galaxies before reionisation: it has been shown this should extend over a physical size of ≈ 1 pMpc[27] and as a result, the diffuse Lyα emission has extremely low surface brightness[117], orders of magnitude lower than observed in the spectrum of JADES-GS-z13-1-LA taken over a solid angle of $0.199'' \times 0.461''$ spanned by a micro-shutter on sky[17]. Moreover, our nodding background subtraction technique would largely self-subtract this signal that is predicted to have approximately uniform central surface brightness[27].

Synthetic photometry and path-loss corrections

To assess the quality of the NIRSpec spectrophotometry and quantify any additional path losses not already accounted for by the data reduction pipeline (see NIRSpec observations and data reduction), we created 'synthetic' NIRSpec photometry to directly compare against the available NIRCam measurements (cf. Extended Data Table 1). Noting all variations of the NIRSpec data reduction yield consistent results, including between the 5-pixel and 3-pixel extractions (Supplementary information), we calculated synthetic flux densities by convolving the sigma-clipped spectrum (with only the default point-source path-loss correction applied; see NIRSpec observations and data reduction) with the NIRCam filter profiles. The corresponding uncertainties were derived using the full covariance matrix and therefore take into account correlated noise properties directly estimated from the measurements. A comparison between the different photometry variants considered here, as derived from NIRSpec (synthetic) and NIRCam (forcepho and CIRC2; see Photometric measurements), is provided in Supplementary Material Fig. 4.

We used the emcee[107] package to perform a linear fit to the ratios of the synthetic NIRSpec photometry to the NIRCam photometry (both the forcepho and CIRC2 measurements) as a function of wavelength. Between forcepho and CIRC2, we consistently find the NIRSpec fluxes are approximately 0.7× those measured by NIRCam (a difference of about 0.4 mag). There is no clear dependence on the observed wavelength: in the forcepho case, the linear fit has a slope of $-0.01 \pm 0.06\,\mu m^{-1}$, and the CIRC2 photometry has $0.00 \pm 0.05\,\mu m^{-1}$. We note that the point-source approximation used by the pipeline to apply the initial path-loss corrections (NIRSpec observations and data reduction) should be valid in the case of JADES-GS-z13-1-LA, as it is essentially unresolved by NIRCam (see Photometric measurements). However, these corrections are subject to a systematic uncertainty on the intra-shutter position introduced by the limited pointing accuracy of the MSA, which is anticipated[17] to be of the order of 25 mas (i.e. comparable to or larger than the size of JADES-GS-z13-1-LA). This effect is enhanced for sources close to the micro-shutter edge (as is the case for JADES-GS-z13-1-LA; Fig. 1), where the path-loss correction shows the largest gradient[61].

UV magnitude and slope

We first measured the absolute UV magnitude M_{UV} and slope β_{UV} directly from the different sets of photometry. To perform an unbiased inference of these parameters, we consider filters redwards of $\lambda_{obs} = 2.0\,\mu m$ (i.e. from F210M on), corresponding to rest-frame wavelengths $\lambda_{emit} \gtrsim 1500$ Å at $z = 13$, thus avoiding the strong spectral break and line (Fig. 1). The results are tabulated Extended Data Table 1. Given the uncertainty of the systemic redshift (as discussed in the Spectral modelling section of the Methods), reported uncertainties on the UV magnitude conservatively take into account a systematic uncertainty of $\Delta z = 0.05$. The UV slope is reported for fits taking into account all available NIRCam filters, covering the rest frame at $\lambda_{emit} \lesssim 3500$ Å. Alternatively, since empirical measurements of UV slopes in the literature use slightly different wavelength ranges (e.g. refs.[31, 118–121]), we also report the UV slope only using NIRCam filters below $\lambda_{obs} \approx 3.5\,\mu m$ (i.e. up to and including F335M), corresponding to $\lambda_{emit} \lesssim 2500$ Å. This reflects the commonly adopted prescription by Calzetti et al.[122] and more closely mimics the fitting range discussed in Spectral modelling.

The UV magnitude inferred from the (uncorrected) NIRSpec measurements is 0.4-0.5 mag fainter than those from NIRCam, reflecting the discrepancy found in Synthetic photometry and path-loss corrections. Based on the CIRC2 photometry, we find a UV magnitude of $M_{UV} = -18.66^{+0.04}_{-0.04}$, in good agreement with $M_{UV} = -18.73 \pm 0.04$ reported by Robertson et al.[16] (corrected for differences in the adopted cosmology) based on Kron[123] photometry. Among the three sets of photometry considered here, our best-fitting UV slopes are consistently very steep ($\beta_{UV} \lesssim -2.7$), again agreeing with $\beta_{UV} = -2.73 \pm 0.13$ found by Robertson et al.[16] when considering the full wavelength range observed by NIRCam ($\lambda_{emit} \lesssim 3500$ Å). We find a considerably steeper slope yet ($\beta_{UV} < -3$) in the wavelength

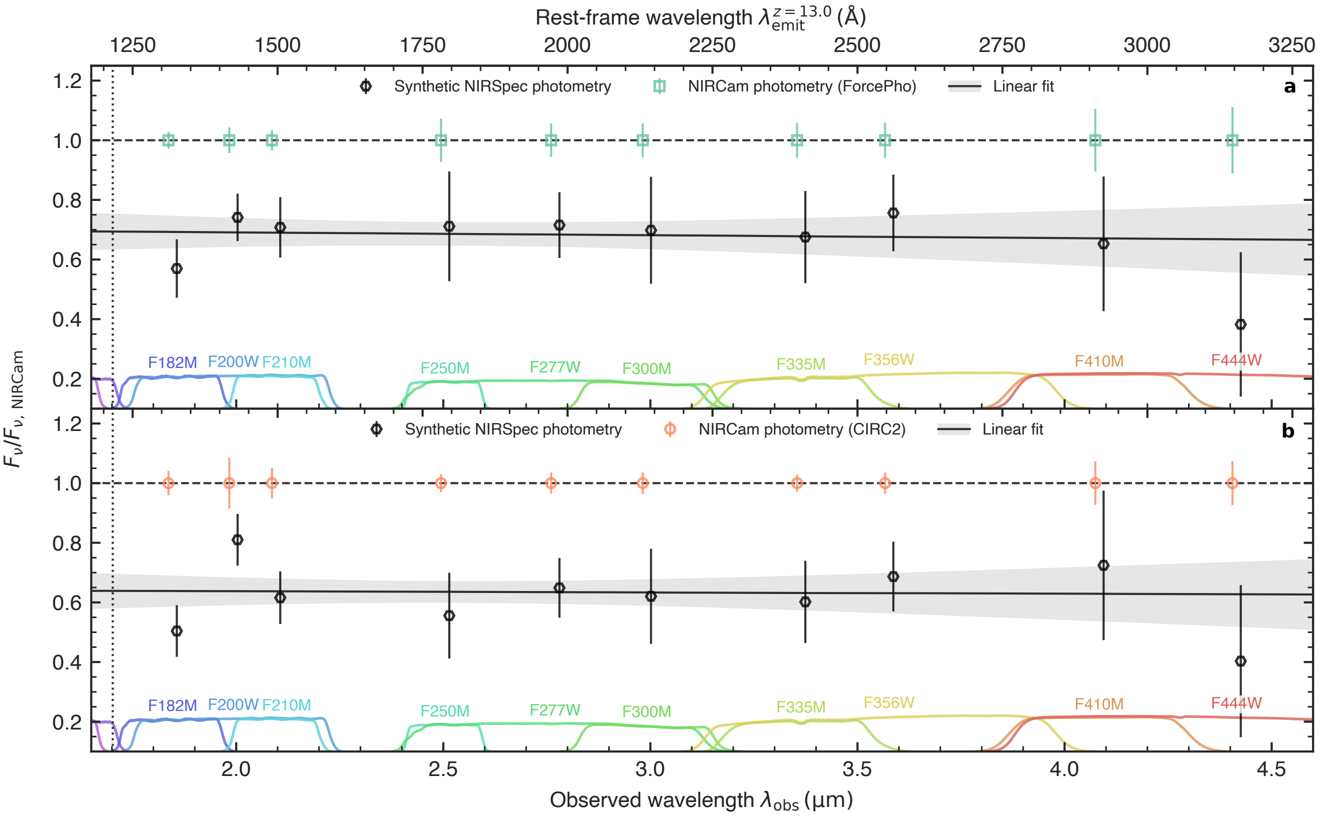

Supplementary Material Fig. 4 | Comparison between NIRCam and NIRSpec (spectro)photometry of JADES-GS-z13-1-LA. Synthetic NIRSpec photometry (see Synthetic photometry and path-loss corrections) is plotted relative to the FORCEPHO (panel **a**) and CIRC2 (panel **b**) photometry in each available NIRCam filter redwards of the observed wavelength of Lyα at $z = 13$ (dotted vertical line).

range up to $\lambda_{emit} \lesssim 2500$ Å (though with larger uncertainties, as a result of the smaller wavelength range and number of data points). Finally, we provide an estimate of the bolometric luminosity (L_{bol}) by integrating the power law with a lower bound at $\lambda_{He\,II} = 227.84$ Å (i.e. the He II ionisation edge at 54.4 eV, where stellar SEDs are typically suppressed[32]).

Stellar population synthesis modelling

We modelled the full SED of JADES-GS-z13-1-LA taking into account NIRCam photometry[★] as well as the NIRSpec/PRISM measurements (the 3-pixel extraction from the default pipeline) with standard SPS models. Specifically, we follow the procedure outlined in Witstok et al.[11], using the Bayesian Analysis of Galaxies for Physical Inference and Parameter EStimation (BAGPIPES[104]) set up with v2.2.1 Binary Population and Spectral Synthesis (BPASS[124]) binary-star models, assuming the default BPASS IMF (stellar mass upper limit of 300 $M_\odot$). We masked the NIRSpec measurements in the spectral region around Lyα, 1150 Å $< \lambda_{emit} <$ 1450 Å, where the complex spectral shape featuring a strong Lyα emission line near a steep turnover cannot be reproduced straightforwardly by standard SED modelling codes such as BAGPIPES, and therefore will be modelled separately in Spectral modelling.

We adopted the non-parametric star formation history (SFH) of Leja et al.[125] with 6 bins in lookback time t. As in Tacchella et al.[126], the first two of these are spaced between 0 Myr $< t <$ 5 Myr and 5 Myr $< t <$ 10 Myr, with logarithmic spacing for the 4 remaining bins up to $z = 20$. We adopted the 'bursty-continuity' prior for the SFR ratio between adjacent time bins[127], having confirmed that consistent results are achieved for a more smoothly varying SFH ('standard continuity'). We varied the total stellar mass formed over $0 < M_* < 10^{15}\,M_\odot$ and stellar metallicity across $0.001\,Z_\odot < Z_* < 1.5\,Z_\odot$, both with log-uniform priors. Our default model includes nebular emission, which BAGPIPES derives from CLOUDY models[106], where the nebular metallicity is fixed to the stellar one. The incident radiation field is set by the relevant SPS models, modulated by a freely varying ionisation parameter ($-3 < \log_{10} U < -0.5$). We included a flexible Charlot & Fall[128] prescription to model the dust attenuation, as detailed in Witstok et al.[11] (see also ref.[129]). The PRISM spectrum was assumed to have a spectral resolution as derived for a uniformly illuminated micro-shutter, noting that fitting only to the continuum (while masking the observed line and break) the precise spectral resolution will not significantly impact the modelled observed spectrum. A first-order polynomial correction to the spectroscopic data[130] was included to account for the mild discrepancy between NIRSpec and NIRCam (Synthetic photometry and path-loss corrections).

★ We have verified that including the MIRI/F770W photometric measurement does not noticeably change our findings, so we chose not to include it in the fitting results shown here unless explicitly mentioned.

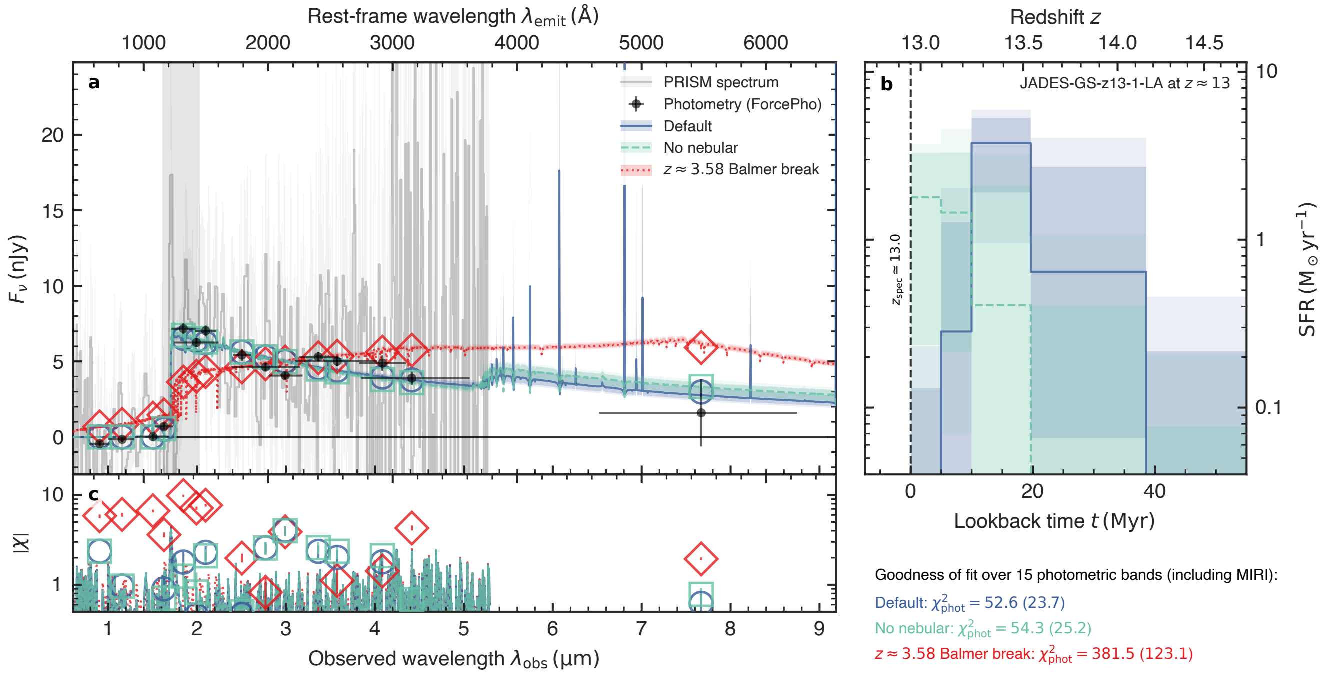

Supplementary Material Fig. 5 | SED modelling of JADES-GS-z13-1-LA. Three different SPS models produced by BAGPIPES are shown: the default setup (circles), a model without nebular emission ('No nebular', squares), and a forced low-redshift model ($z \approx 3.58$ Balmer break, diamonds; see Stellar population synthesis modelling for details). **a**, Observed NIRCam (FORCEPHO) and MIRI photometry (black points) and NIRSpec/PRISM spectrum (grey line; smoothed by a 3-pixel median filter for visualisation), overlaid by the three models (lines according to the legend in the top right and open symbols). Error bars on the photometry and light-grey shading around the spectrum represent 1σ uncertainties; darker (lighter) shading around the model lines shows 1σ (2σ) variation in the posterior distribution. **b**, Residuals between the observations and modelled spectra and photometry expressed in terms of the absolute value of χ, the difference normalised by 1σ observational uncertainty. Goodness-of-fit statistics in the form of χ^2 calculated over the 15 photometric bands (including the MIRI/F770W measurement) are annotated (conservative estimates in brackets). **c**, Inferred SFH in the two $z \approx 13$ models (default and without nebular emission; shading as in panel a).

In our default model, we allow the redshift to vary within an interval of $\Delta z = 0.1$ centred on $z = 13.0$, so that we may explicitly marginalise over the uncertainty of the systemic redshift (further discussed in Spectral modelling). In addition, we considered a variation of the default model where we explicitly turn off nebular emission: this would be the case if the galaxy has a LyC escape fraction close to $f_{\rm esc,\,LyC} = 100\%$. The resulting best-fitting models are shown in Supplementary Material Fig. 5 and summarised in Supplementary Material Table 1.

Also shown in Supplementary Material Fig. 5 is an alternative ('Balmer-break') model forced to be at lower redshift by centring the $\Delta z = 0.1$ interval on $z = 3.583$ instead, in which case the observed spectral break would correspond to a Balmer break and the (in this case not masked) observed emission line would be [O II] (see Emission-line properties). When fitting this model, we include the MIRI/F770W photometric point. In agreement with Hainline et al.[15] and Robertson et al.[16], we find this low-redshift solution produces a poor fit to the observed SED of JADES-GS-z13-1-LA ($\chi^2 = 381.5$ over 15 NIRCam and MIRI bands), whereas the data are reproduced much better by a model galaxy at $z \approx 13.0$ instead ($\chi^2 = 52.6$ in the default model).

Even so, the comparatively high χ^2 (with $p = 4.5 \times 10^{-6}$ for the default model) indicates that photometric errors are potentially underestimated, or that the standard SPS models fall short of accurately describing the data. While the FORCEPHO photometry circumvents the correlated noise between pixels in the mosaic images, it may still suffer from a degree of systematic uncertainty, including from any imperfections in the sky background subtraction. This effect is taken into account empirically by the aperture photometry (CIRC2), where the estimated uncertainty considers the scatter found in a number of randomly placed empty apertures, a detailed discussion of which can be found in Rieke et al.[55]. The true statistical errors therefore likely consist of a combination of the nominal uncertainty estimated as part of the FORCEPHO modelling and CIRC2 aperture photometry (which covers approximately 70% of the encircled energy of the F444W PSF). When we conservatively combine the estimated FORCEPHO and CIRC2 photometric uncertainties (treating them as statistically independent) to better account for systematic effects, we instead find a more reasonable $\chi^2 = 23.7$ ($p = 0.070$).

Interestingly, we find the goodness of fit in the absence of nebular emission ($\chi^2 = 54.3$ or more conservatively $\chi^2 = 25.2$) to be essentially the same as in the default model, even if the inferred SFHs vary substantially between these models: the SFH in the model without nebular emission steadily increases, whereas the default model prefers an SFH that rises initially, but subsequently declines over the last 10 Myr. We conclude this downturn in SFH must be artificial, and only preferred by the default model in order to reproduce the very steep UV slope of $\beta_{\rm UV} \lesssim -2.7$ independently measured both by NIRCam and NIRSpec (see UV magnitude and slope), as this requires reducing the number of very young (< 10 Myr) OB-type stars, which produce a large number of ionising photons at fixed UV luminosity[32], thus minimising the contribution of nebular-continuum

Supplementary Material Table 1 | Stellar properties of JADES-GS-z13-1-LA.

Quantity	BAGPIPES		BEAGLE
	Default	No nebular	$f_{\rm esc,\,LyC} \neq 0$
M_* (10^7 $M_\odot$)	$5.5^{+2.1}_{-1.2}$	$2.7^{+1.8}_{-0.7}$	$2.6^{+3.5}_{-1.5}$
Σ_* (10^3 $M_\odot$ pc^{-2})	$> 7.1^{+2.8}_{-1.6}$	$> 3.5^{+2.3}_{-0.9}$	$> 3.3^{+4.6}_{-2.0}$
Z_* ($Z_\odot$)	$0.3^{+0.8}_{-0.1}$ %	$0.6^{+1.1}_{-0.3}$ %	2^{+10}_{-1} %
SFR_{10} ($M_\odot$ yr^{-1})	$0.19^{+0.51}_{-0.17}$	$1.84^{+0.55}_{-0.62}$	$0.67^{+0.36}_{-0.18}$
$\Sigma_{\rm SFR}$ ($M_\odot$ yr^{-1} kpc^{-2})	$> 25^{+66}_{-21}$	$> 240^{+71}_{-81}$	$> 87^{+47}_{-23}$
t_* (Myr)	22^{+9}_{-6}	11^{+13}_{-6}	21^{+38}_{-15}
A_V (mag)	$0.04^{+0.04}_{-0.03}$	$0.11^{+0.08}_{-0.07}$	$0.08^{+0.19}_{-0.05}$
$\log_{10} U$	$-1.91^{+0.82}_{-0.79}$	–	$-2.59^{+0.91}_{-0.86}$
$f_{\rm esc,\,LyC}$	0^*	1^*	$0.81^{+0.14}_{-0.32}$

Best-fitting parameters, taken as the median of their marginalised posterior, are shown for the BAGPIPES and BEAGLE models with different assumptions on nebular emission (see Stellar population synthesis modelling). Error bars represent a 1σ uncertainty (16th and 84th percentiles). Rows: stellar mass (M_*) in 10^7 Solar masses, stellar mass surface density (Σ_*) in 10^3 Solar masses per square parsec, stellar metallicity (Z_*) in units of Solar metallicity, star formation rate in Solar masses per year averaged on a timescale of 10 Myr (SFR_{10}), its corresponding surface density ($\Sigma_{\rm SFR}$) in Solar masses per year per square kiloparsec, mass-weighted stellar age (t_*) in Myr, visual dust extinction (A_V) in magnitudes, ionisation parameter U (if applicable), and ionising-photon escape fraction ($f_{\rm esc,\,LyC}$).
* Value is fixed in this model.

emission (which typically has $\beta_{\rm UV} \approx -2$; refs. [29, 131]) that drives the UV slope upwards [119–121,132].

The tendency towards a high LyC escape fraction is independently confirmed by BEAGLE (BayEsian Analysis of GaLaxy sEds [105]) modelling with varying $f_{\rm esc,\,LyC}$, largely as described in Curtis-Lake et al. [3]. Briefly, we fitted the NIRSpec/PRISM spectrum only, again masking the spectral region around Lyα, 1150 Å $< \lambda_{\rm emit} <$ 1450 Å. We assumed a constant SFH and a Chabrier [133] IMF with stellar mass upper limit of 300 $M_\odot$. Reassuringly, the best-fitting parameters (Supplementary Material Table 1) are in good agreement with the BAGPIPES results despite different approaches, particularly considering the absence of normalisation to the NIRCam photometry in the BEAGLE fit. The BEAGLE fit yields a posterior $f_{\rm esc,\,LyC} = 0.81^{+0.14}_{-0.32}$ (median and 16th-84th percentiles).

References (continued)

113. Brammer G. B., van Dokkum P. G., Coppi P., 2008. EAZY: A Fast, Public Photometric Redshift Code, ApJ, 686, 1503

114. Curti M., et al., 2024. JADES: The star-formation and chemical enrichment history of a luminous galaxy at $z \sim 9.43$ probed by ultra-deep JWST/NIRSpec spectroscopy, p. arXiv:2407.02575 arXiv:2407.02575

115. Li Y., Leja J., Johnson B. D., Tacchella S., Naidu R. P., 2024. No Top-heavy Stellar Initial Mass Function Needed: The Ionizing Radiation of GS9422 Can Be Powered by a Mixture of an Active Galactic Nucleus and Stars, ApJ, 969, L5

116. Shapley A. E., et al., 2015. The MOSDEF Survey: Excitation Properties of z ∼ 2.3 Star-forming Galaxies, ApJ, 801, 88

117. Padmanabhan H., Loeb A., 2024. Intergalactic Lyman-α haloes before reionization are detectable with JWST, J. Cosmology Astropart. Phys., 2024, 059

118. Cullen F., et al., 2023. The ultraviolet continuum slopes (β) of galaxies at $z \simeq 8-16$ from JWST and ground-based near-infrared imaging, MNRAS, 520, 14

119. Topping M. W., et al., 2024. The UV continuum slopes of early star-forming galaxies in JADES, MNRAS, 529, 4087

120. Cullen F., et al., 2024. The ultraviolet continuum slopes of high-redshift galaxies: evidence for the emergence of dust-free stellar populations at z > 10, MNRAS, 531, 997

121. Austin D., et al., 2024. EPOCHS III: Unbiased UV continuum slopes at 6.5<z<13 from combined PEARLS GTO and public JWST NIRCam imaging, p. arXiv:2404.10751 arXiv:2404.10751

122. Calzetti D., Kinney A. L., Storchi-Bergmann T., 1994. Dust Extinction of the Stellar Continua in Starburst Galaxies: The Ultraviolet and Optical Extinction Law, ApJ, 429, 582

123. Kron R. G., 1980. Photometry of a complete sample of faint galaxies., ApJS, 43, 305

124. Eldridge J. J., et al., 2017. Binary Population and Spectral Synthesis Version 2.1: Construction, Observational Verification, and New Results, Publ. Astron. Soc. Australia, 34, e058

125. Leja J., Carnall A. C., Johnson B. D., Conroy C., Speagle J. S., 2019. How to Measure Galaxy Star Formation Histories. II. Nonparametric Models, ApJ, 876, 3

126. Tacchella S., et al., 2023. JWST NIRCam + NIRSpec: interstellar medium and stellar populations of young galaxies with rising star formation and evolving gas reservoirs, MNRAS, 522, 6236

127. Tacchella S., et al., 2022. On the Stellar Populations of Galaxies at z = 9-11: The Growth of Metals and Stellar Mass at Early Times, ApJ, 927, 170

128. Charlot S., Fall S. M., 2000. A Simple Model for the Absorption of Starlight by Dust in Galaxies, ApJ, 539, 718

129. Chevallard J., et al., 2019. Simulating and interpreting deep observations in the Hubble Ultra Deep Field with the JWST/NIRSpec low-resolution 'prism', MNRAS, 483, 2621

130. Carnall A. C., et al., 2019. The VANDELS survey: the star-formation histories of massive quiescent galaxies at 1.0 < z < 1.3, MNRAS, 490, 417

131. Saxena A., et al., 2024. Hitting the slopes: A spectroscopic view of UV continuum slopes of galaxies reveals a reddening at z > 9.5, p. arXiv:2411.14532 arXiv:2411.14532

132. Byler N., Dalcanton J. J., Conroy C., Johnson B. D., 2017. Nebular Continuum and Line Emission in Stellar Population Synthesis Models, ApJ, 840, 44

133. Chabrier G., 2003. Galactic Stellar and Substellar Initial Mass Function, PASP, 115, 763
